# Dysfunctional voiding: the importance of non-invasive urodynamics in diagnosis and treatment

**DOI:** 10.1007/s00467-017-3679-3

**Published:** 2017-05-31

**Authors:** Joanna C. Clothier, Anne J. Wright

**Affiliations:** Department of Paediatric Nephro-urology, Evelina London Children’s Hospital, Guy’s and St Thomas’ NHS Foundation Trust, Westminster Bridge Rd, SE1 7EH London, UK

**Keywords:** Dysfunctional voiding, Lower urinary tract dysfunction, Urinary tract infection, Uroflowmetry EMG, Pelvic floor

## Abstract

In Dysfunctional voiding, failure of the external sphincter-pelvic floor complex to relax during micturition results in bladder outflow obstruction with a spectrum of presentation from more benign lower urinary tract dysfunction including recurrent urinary tract infections, to significant upper tract pathology and end-stage renal failure. There is no underlying neurological or anatomical cause and the condition is postulated to be a largely learnt behavior. Diagnosis relies on non-invasive urodynamics and in particular uroflowmetry, plus or minus EMG, which is also used in biofeedback, the mainstay of treatment. The etiology, presentation, diagnosis, and treatment with particular emphasis on non-invasive urodynamics are covered.

## Introduction

Dysfunctional voiding (DV), or micturition that is obstructed due to failure of the external urethral sphincter-pelvic floor complex to relax appropriately (in the absence of any neuroanatomical abnormality), has the highest rates of urinary tract infection (UTI), urinary incontinence (UI), vesicoureteric reflux (VUR), and bowel dysfunction of any functional lower urinary tract disorder and similar rates of frequency and urgency to overactive bladder (OAB) [1]. Whilst a very careful history may point to the primary problem, it is often the secondary issues of UI and UTI that are the presenting complaints. Treatment is often suboptimal and the child continues to suffer with potentially harmful consequences. This article reviews the condition of DV including presentation, diagnosis, and treatment with a particular emphasis on the important role of non-invasive urodynamics.

## Normal childhood bladder function

Neurological control of the bladder governs two basic phases:

1. *Storage phase* facilitated by hypogastric plexus stimulation (T10-L2) resulting in sympathetic relaxation of the detrusor muscle and stimulation of the urethral sphincter. Adequate compliance of the bladder wall allows storage of urine at low bladder pressures (allowing filling from the upper tracts).
2. *Voiding or micturition phase* where parasympathetic stimulation from the sacral micturition center (S2-S4) via the pelvic nerve facilitates detrusor contraction and internal sphincter relaxation, and somatic input via the pudendal nerve relaxes the external urethral sphincter. This allows the lowest possible detrusor pressure able to overcome outflow resistance during voiding with complete emptying of the bladder.

Smooth coordination between the two phases of storage and micturition depends on intact neural pathways in the central nervous system in the cortical, pontine, and sacral micturition centers, which allow social planning and timing, coordination, and amplification, respectively.

The bladder is an unusual autonomic visceral organ in that it interfaces with a voluntarily controlled somatic sphincter. The rectum is similar. Once toilet-training is achieved, voluntary initiation of voiding over a wide range of bladder volumes can be initiated at will in an “on-off” manner facilitated via a series of positive feedback loops using supraspinal pathways. The initial concept that babies micturate via a reflex spinal pathway is no longer held with evidence that neonates are aware of voiding with involvement of CNS centers [2, 3] and later development of voluntary control. Neonatal voiding is however characterized by small frequent voids every 1–2 h with an immature pattern of interrupted detrusor-sphincter dyscoordinated voiding seen in 30% of cases with associated incomplete bladder emptying. This immature pattern improves and resolves by the time of toilet training [4]. By 5 years of age, children should be continent both by day and by night with increasing bladder volumes (expected bladder capacity EBC = (age/years + 1) × 30 ml, from 4 to 12 years) and normal urinary frequency of 4–7 times/day [5]. Lower urinary tract dysfunction (LUTD), usually manifested as nocturnal enuresis (NE) and/or daytime urinary incontinence (DUI) together with the common associations of constipation, fecal incontinence, and UTI, results in failure to attain normal continence patterns. A recent study of children over the age of 4 years presenting with UTI showed that 59% had LUTD (90% and 60% of these suffering from DUI and NE, respectively) compared with 2% of controls, and 37% had significant impairment in quality of life compared with 0% of controls [6].

By far the commonest condition causing LUTD in children is overactive bladder (OAB) associated with uninhibited detrusor contractions during the storage phase of bladder function on invasive urodynamics. The classic symptom is that of urgency with additional holding maneuvers, urge incontinence, urinary frequency, DUI, and NE. The voiding phase is not generally implicated.

DV on the other hand is dysfunction of the sphincter during voiding and refers only to the voiding phase in anatomically and neurologically intact patients. Simultaneous contraction of the sphincter and detrusor in the presence of a neurological condition is described as detrusor sphincter dyssynergia. These two terms are not interchangeable, as whilst the dyscoordination between detrusor and sphincter may be similar the underlying etiology is not [5].

## Definition, epidemiology, and pathogenesis

Beer first described voiding difficulty, recurrent infections, and sphincteric incoordination in neurologically normal children in 1915 [7]. Hinman recognized a severe form of obstructed voiding with upper tract deterioration and psychological associations [8] and Allen first studied the urodynamic characteristics and used the term DV [9]. A number of other terms have been used to describe DV: non-neurogenic neurogenic bladder, Hinman syndrome, Hinman–Allen syndrome, occult neurogenic bladder, detrusor sphincter dyscoordination, dysfunctional elimination syndrome, and learned voiding dysfunction.

The current definition of dysfunctional voiding given by the International Children’s Continence Society (ICCS) [5] is:
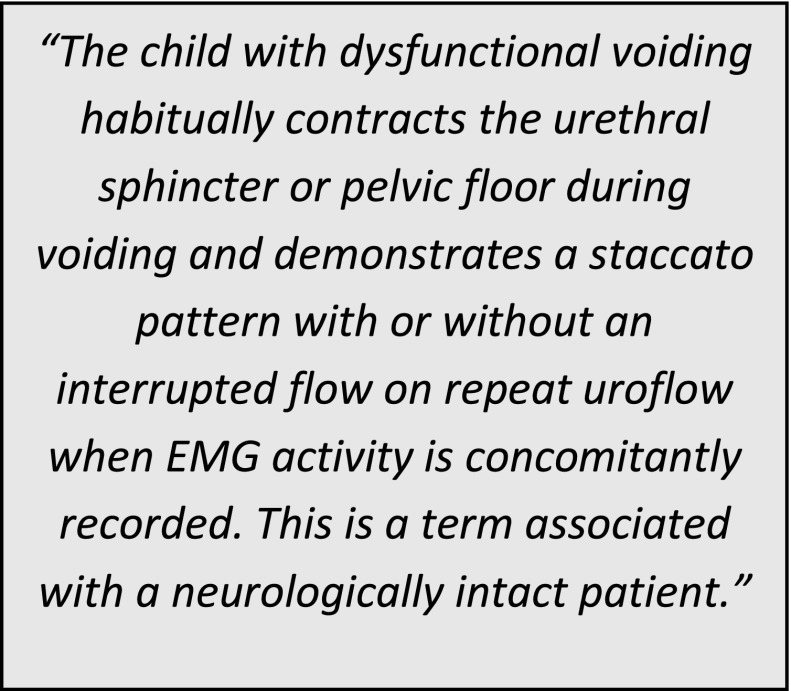


The true estimate of DV *prevalence* in the general population is not known because of diagnostic failure, overshadowing by storage symptoms and recurrent urinary tract infections, and confusion with terminology. Reliable surveys in children with wetting problems have reported anything from 4.2 to 32% [10].

The causes of dysfunctional voiding are likely to be multi-factorial.

### Congenital or genetic

A number of authors have reported congenital syndromes presenting with severe obstructed voiding in the absence of neuroanatomical abnormalities and associated upper tract deterioration. The early and severe presentation (in some cases antenatally), rules out learnt maladaptation and points to congenital or genetic causes possibly associated with subclinical changes in the pons and pontine micturition center [11–13]. DV has been described in males with Down’s syndrome [14]. Ochoa syndrome (urofacial syndrome) is a rare condition characterized by congenital urinary bladder dysfunction, obstructed voiding, frequent VUR, and upper tract dilatation/damage together with constipation in association with an abnormal grimace upon smiling, laughing, and crying. Ochoa first reported the autosomal recessive association of urofacial syndrome with a probable mapping to gene 10q23-q24 [15]. Recently, it has been shown that null mutations of HPSE2, or less commonly LRIG genes, code for proteins that work in a common pathway to facilitate neural growth into the bladder [16]. Currently, congenital and genetic causes are perceived to be uncommon. Further genetic studies may change this perception in the future.

### Persistent immature infantile patterns of dyscoordinated voiding

There is increasing evidence that ease of diaper use and changes in parenting practice in the developed world has delayed toilet training age to later childhood and this may be facilitating the perpetuation of immature infantile bladder patterns with persistent urinary symptoms. A large, powerful, longitudinal prospective population-based study from the Avon region in the United Kingdom (long-term follow-up of approximately 14,000 children born in 1991/1992) showed that the children of parents who initiated toilet training after 24 months of age (considered to be late toilet training) were more likely to experience the following than those who initiated toilet training between 15–24 months:

- Delayed acquisition of daytime bladder control (*p* < 0.001)
- Persistent daytime wetting (*p* < 0.001)
- A relapse in daytime wetting (*p* < 0.001) following a period of previously being dry

Mothers who were older and with more educational qualifications and mothers who felt that their child was developmentally delayed and/or had a more difficult temperament started toilet training later. The authors surmise that delaying onset of toilet training until after 2 years of age prolongs the exposure time to potential stressors that can interfere with acquisition of bladder control. A limitation of the study was that there was no information available about the methods of toilet training used or the type of daytime wetting experienced subsequently [17].

A small Swedish study longitudinally followed 59 children with non-invasive bladder monitoring from zero to 6 years and demonstrated acquisition of bladder control for day and night-time respectively at ages 3.5 and 4 years, together with later commencement in toilet training. Awareness of voiding however was noted from 18 months of age and the authors suggest that this is the opportune time to commence training. Children with a small post-void residual at 6 months of age and a large bladder capacity were more likely to become dry by day earlier [4].

Using similar study methods to the Swedish study, 47 Vietnamese children had bladder assessments from zero to 3 years of age in association with early assisted toilet training and were compared with the Swedish cohort. Ninety-eight percent of Vietnamese children were toilet trained by 2 years of age in contrast to only 5% of Swedish children having commenced training. In addition, the study demonstrated resolution of dyscoordination between detrusor and sphincter muscles with minimal to no post-void residual urine by 9 months of age in the Vietnamese children compared to 36 months of age in Swedish children, suggesting that early toilet training confers protective bladder factors [18].

### Learned or acquired pelvic floor-sphincteric behavior

It is thought that DV develops due to learnt and habitual dysfunction of the pelvic floor and that this is probably the commonest cause of the condition. There are many potential triggers or reasons why a child may tense their pelvic floor and engage their external urethral sphincter voluntarily during voiding; difficult toilet training in association with difficult child temperament and control issues, painful urination caused by urinary tract infection or local irritation in association with vulvitis or balanitis, dislike of urination in strange places outside of the home, fear of unclean toilets, social embarrassment and needing to eliminate only within a safe, sound-proof, personal space like home. Some reports suggest that DV is related to emotional and psychosocial problems or maturational delay and there are reports of patients developing dysfunctional voiding following childhood sexual abuse [19].

Certain medical conditions are associated with DV. It is not uncommon for the child with urge incontinence to develop an overactive and high tone pelvic floor in response to recurrent detrusor overactive contractions in an attempt to avoid wetting, although there is no evidence that OAB necessarily evolves into DV with time [20]. Likewise, dysuria with UTI may result in pelvic floor activity during voiding that may become habitual, however, a study in children diagnosed with UTI or VUR before the age of 2 years, found no differences in dysfunctional voiding scores compared with controls [21]. Increasingly, DV is recognized as a cause of recurrent UTI particularly in girls with a recent recommendation to screen for bladder dysfunction [22, 23]. There is a strong association between bladder dysfunction and constipation and certainly, it follows that dislike of urination with an increased pelvic floor tone may well co-affect the external anal sphincter and inhibit rectal activity resulting in constipation. Sixty-three percent of children with DV fulfill the criteria for functional constipation more than those with urge incontinence and other urological disorders [24]. There is a known association between LUTD and VUR. Silva et al. conducted a multivariate analysis on 506 children managed conservatively for VUR and found only four variables at presentation that were predictive of VUR resolution: non-white race, mild grade of VUR, absence of renal damage and absence of dysfunctional voiding [25]. American guidelines recommend any child over the age of 1 year with VUR and clinical evidence of bladder bowel dysfunction (BBD) undergo evaluation and treatment for BBD as an integral part of VUR therapy [26]. Following a retrospective review of patients with lower urinary tract symptoms, OAB plus DV and DV were major risk factors for VUR, UTI, and renal damage. The presence of VUR in children with LUTD plays an important role with regard to UTI and renal damage, with dilating VUR a major risk factor associated with renal damage [27]. Whether reflux is secondary to LUTD, vice versa, or coincidental, is controversial. VUR associated with LUTD has been shown to resolve with targeted LUTD treatment in 45% of cases; resolution rates were similar for all grades of reflux. Patients with dysfunctional voiding had the most improvement and greatest resolution of reflux [28].

## History, presentation, and examination

**Fig. 1:**
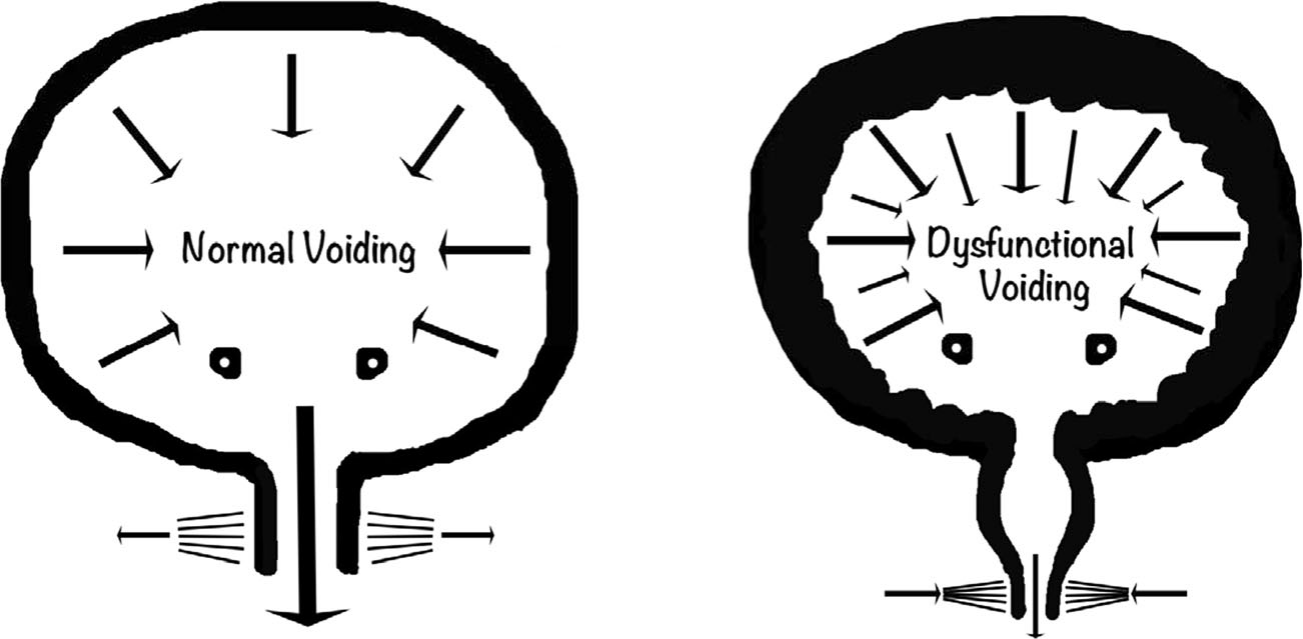
Normal co-ordinated voiding (*left*) with relaxation of external urethral sphincter and lower voiding detrusor pressure compared with dysfunctional voiding (*right*) with external sphincter contraction during voiding, dilatation of the proximal urethra (*spinning top*), detrusor hypertrophy and raised voiding detrusor pressure. Image courtesy of Mr. Massimo Garriboli

**Fig. 2:**
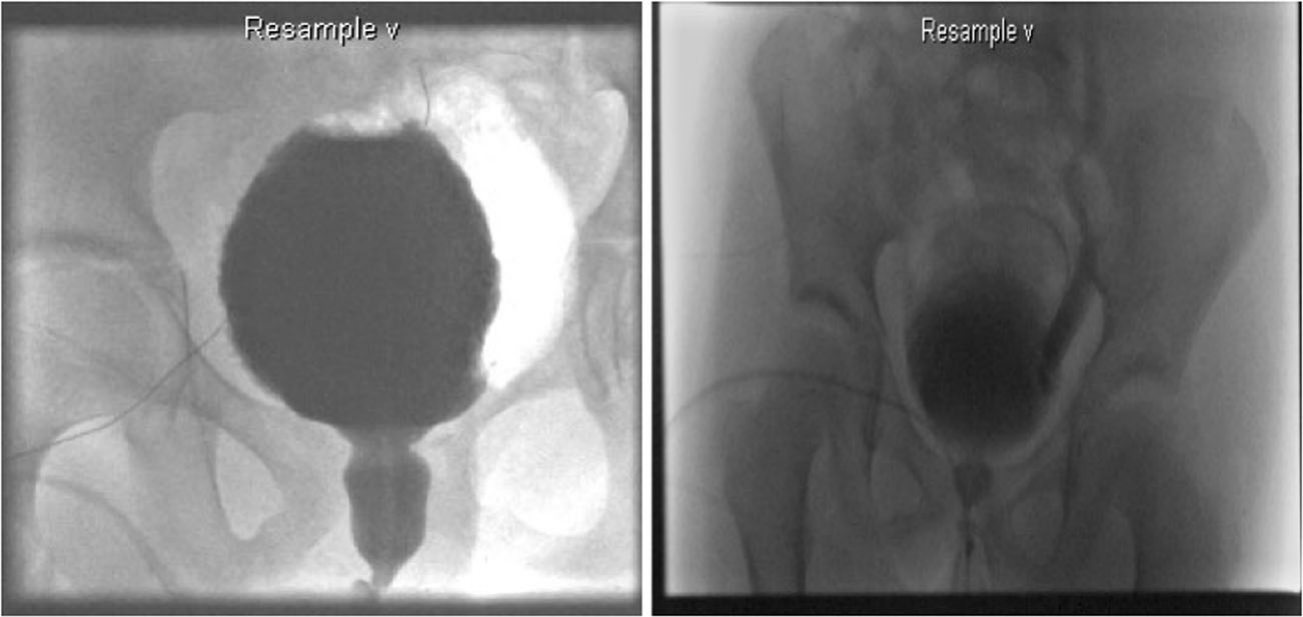
Fluoroscopy showing spinning top urethra, trabeculation, and vesicoureteric reflux in dysfunctional voiding

Dysfunctional voiding can present at any age but most typically after toilet training and before puberty with recurrent urinary tract infections, day or night-time urinary incontinence, difficulties with voiding or urethral/suprapubic pain. The condition has a broad spectrum of presentation and outcomes from relatively harmless to the life-threatening complication of end-stage renal failure (ERF). The florid form presents similarly to the classical neuropathic or posterior urethral valve bladder with associated bilateral hydronephrosis and ERF. Bladder outflow obstruction (BOO) caused by the urethral sphincter may result in a number of physical changes in the urinary tract from distal to proximal with associated symptoms and signs; see Table 1 and Figs. 1 and 2.

**Table 1:** Symptoms and signs that can be observed in dysfunctional voiding

Obstructive level (distal to proximal)	Symptoms	Signs
1. External urethral sphincter	Voiding symptoms • Difficulty with initiating or maintaining stream • Abnormal stream	**Physical**: observed interrupted or varying urinary stream **Urodynamics**: staccato, interrupted uroflow/voiding EMG activity **Radiology**: spinning top urethra on fluoroscopy
2. Proximal urethra	Urethral (usually penile) pain during voiding due to abnormal flow Hematuria	**Physical**: urethritis on cystoscopy **Radiology**: spinning top urethra
3. Detrusor –compensatory hypertrophy Detrusor overactivity Decreased detrusor compliance Decreased bladder capacity	Storage symptoms: • Urgency • Urge incontinence • Frequency	**Physical:** evidence of incontinence, holding manoeuvres **Urodynamics:** Small MVV from bladder diary Detrusor overactivity and/or low compliance on invasive urodynamics **Radiology:** thickened bladder wall, trabeculation, diverticulae
4. Detrusor –myogenic decompensation/failure	Voiding symptoms • Difficulties with initiating and maintaining void • Infrequent voiding • Abdominal straining • Sitting to void in boys • Unusual voiding e.g., situation-specific, only in the bath • Urinary retention	**Physical:** Palpable bladder with no sensation of need to void. Abdominal straining during observed voiding **Urodynamics:** infrequent voiding/large MVV on bladder diary Interrupted uroflow. Abdominal EMG activity during voiding. **Radiology:** Distended large volume bladder on ultrasound
5. Vesicoureteric reflux	Urinary tract infection +/− loin pain/pyelonephritis	**Radiology:** Dilated ureter, urothelial thickening, hydronephrosis, post-void residual on ultrasound. VUR on fluoroscopy. Pseudo post-void residual due to VUR on fluoroscopy. **Nuclear medicine**: VUR on indirect cystogram
6. Renal damage	Poor growth, polydipsia, polyuria	**Physical:** uremia, hypertension **Laboratory**: raised serum creatinine plus other ERF markers **Radiology:** renal scarring, hydronephrosis
Other findings	Symptoms	Signs
7. Incomplete bladder emptying	Sensation of incomplete emptying at end of void Need to return shortly after voiding to try again Significant leakage shortly after voiding	**Physical:** Passage of significant volume of urine on double void **Radiology:** Evidence of post void residual on ultrasound. Incomplete bladder emptying fluoroscopy. **Nuclear medicine**: incomplete bladder emptying on indirect cystogram
8. Asymptomatic bacteriuria	Urinary odor (distinctive)	**Physical:** Child well **Laboratory:** urine dipstick may be positive for white cells, nitrites, blood. Microscopy may show bacterial growth but insignificant white cells.
9. Urinary tract infection	Dysuria, frequency, urgency, smelly urine, abdominal or loin pain, systemic features of being unwell	**Laboratory:** Urine dipstick and urine microscopy, culture suggestive of infection

BOO associated with DV results in voiding difficulty, but confusingly, children most commonly present with secondarily induced storage symptoms including urinary urgency and frequency and urge incontinence. These symptoms may be the only presenting features and typically result from detrusor overactivity, urinary infections or reduced bladder capacity consequent to large residual volumes or compensatory detrusor hypertrophy resulting in a functionally small capacity bladder. Urodynamic studies are required to distinguish between them. Others present with a history of recurrent urinary tract infection or evidence of incomplete bladder emptying on ultrasound scan. Recurrent UTI can occur due to incomplete emptying, trabeculation, or diverticulae of the bladder and VUR.

Around half of children present with voiding difficulty and some present with unexplained urinary retention. They may describe difficulty with voiding in public places, delay in initiating urinary stream, infrequent voiding or requirement of visual, audible, or physical signals (such as voiding in the bath) to allow relaxation and micturition. Adults are more likely to present with actual voiding symptoms than children both due to expressive abilities and the increased prevalence of urinary retentive features in adults.

Detrusor underactivity and acontractility may occur as an end point in a spectrum of storage abnormality in relation to DV commencing with initial reactive high detrusor pressures with subsequent detrusor decompensation resulting in low pressure retention. Voiding is accomplished by straining to increase abdominal pressure in order to initiate and maintain voiding. Symptoms and signs include infrequent voiding ≤3×/day (normal 4-7×/day), large-volume post-void residuals with overflow incontinence and UTI. Children with an over-distended bladder may be continent or have episodes of hesitancy, urge incontinence, or overflow incontinence related to increased intra-abdominal pressure. They tend to have dampness rather than a soaking of clothes and the diary will show infrequent spontaneous voiding of perhaps once or twice per day.

The most severe form of DV is often called Hinman–Allen syndrome or non-neurogenic neurogenic bladder. These patients are found to have a trabeculated and irregularly shaped bladder with high voiding pressures, bilateral high-grade VUR, and a large residual urine volume. This can lead to recurrent UTI, renal scarring, hypertension, and progressive renal failure [8].

Fowler’s syndrome is typically seen in young women aged 20 to 35 years, presenting with urinary retention with increased urethral sphincter tone with polycystic ovarian disease. Often there is a common natural history of relatively mild voiding dysfunction such as infrequent voiding or an intermittent stream. The first retention episode usually occurs following a triggering event such as an operation or childbirth. Investigations reveal abnormal sphincters, as assessed by EMG, transvaginal ultrasonographic volume and urethral pressure profile, with increased sphincter volume and concentric needle EMG demonstration of abnormal decelerating bursts and complex repetitive discharges. Whether Fowler’s syndrome represents a distinct cause of urinary retention or results from a maladaptive behavior and is similar to DV is unclear [29]. Sacral neuromodulation has been shown to restore normal voiding [30].

History taking should be targeted at symptoms of both the storage and voiding phases with particular emphasis on any difficulties with initiating or maintaining urinary stream and any toileting avoidance behaviors. Features not commonly associated with OAB alone should raise suspicions of DV including features of hesitancy, obstructive urinary stream, normal-to-large capacity MVV, evidence of incomplete bladder emptying, and recurrent UTIs. It is vital not to forget a detailed bowel history and to exclude constipation based on Rome IV criteria [31], as well as excluding neurological or anatomical causes. Urethral meatal deformities in girls can have an anterior deflected urinary stream and they cannot void in the ideal toileting position with associated DV and associated treatment failure. Surgical meatal correction may resolve symptoms in 45% [32].

## Investigation and diagnosis

Tools of investigation including bladder and bowel diaries, questionnaires, urinalysis, and ultrasound of the urinary tract (pre-and post-void) should be routinely carried out [33]. Ultrasound is particularly useful for potential evidence of upper tract damage, VUR, bladder abnormalities including thickened bladder wall, diverticulae, urinary sediment, abnormal bladder neck, and rectal diameter (>3 cm suggestive of constipation). The bladder wall is often found to be thickened in those with DV not unlike those with outlet obstruction and measurements using both transabdominal and perineal approach have been described with potential use of bladder wall thickness as a non-invasive tool for evaluation although this requires further study [34]. Urinary vaginal reflux during micturition, presenting with post-void dribble, in particular can be seen on post-void ultrasound and it may mimic DV with low-flow, prolonged, or fractionated uroflowmetry and positive urinalysis (asymptomatic bacteriuria).

### Non-invasive urodynamics: uroflowmetry and pelvic floor EMG

The key to diagnosis of DV is evidence of obstructed voiding during uroflowmetry preferably with accompanying pelvic floor EMG.

**Fig. 3:**
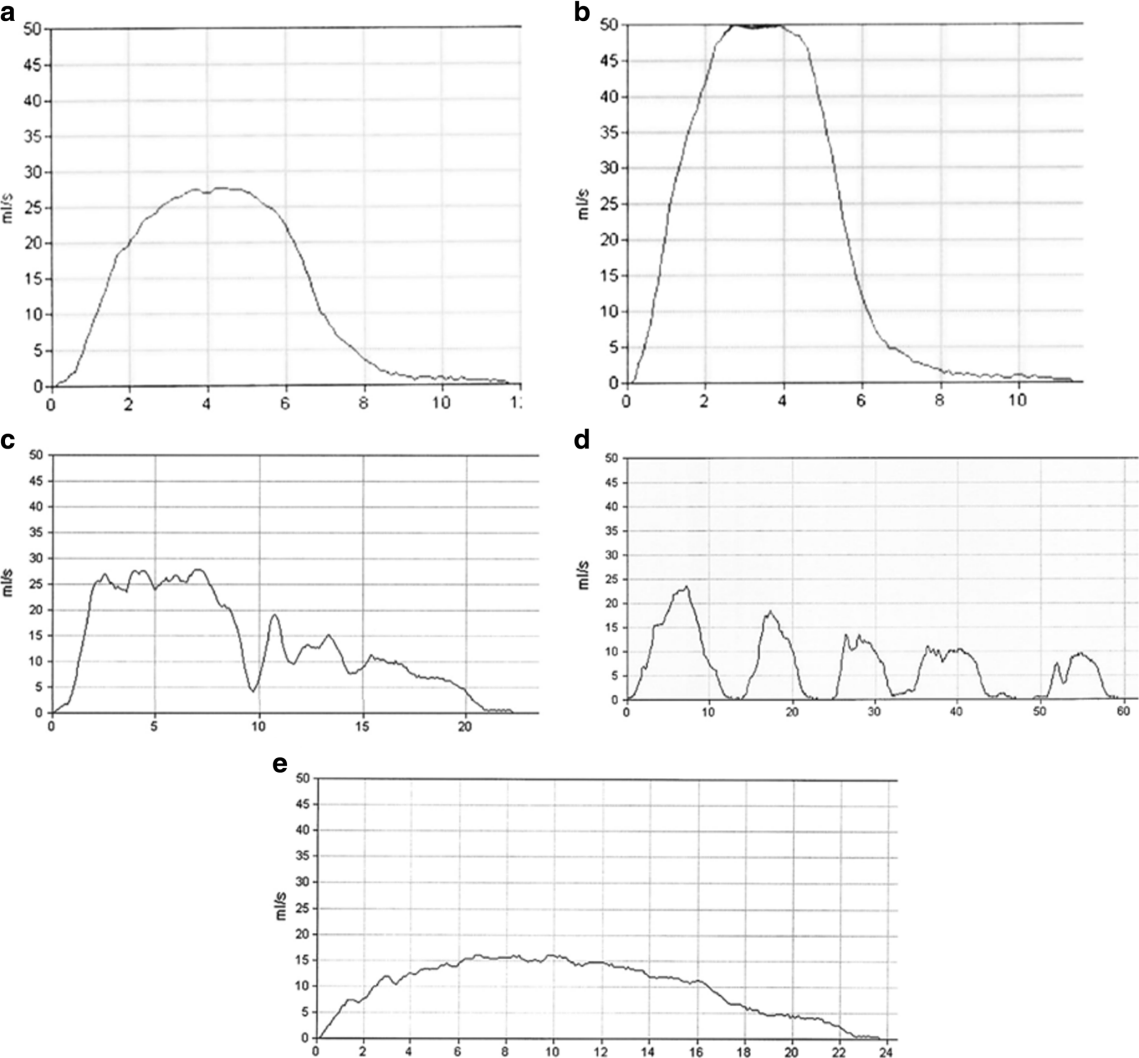
Uroflow curve patterns.** a** Bell-shaped (normal).** b** Tower-shaped (suggestive of overactive bladder).** c** Staccato-shaped (suggests dysfunctional voiding).** d** Interrupted-shaped (suggests underactive detrusor with abdominal straining).** e** Plateau-shaped (suggestive of static bladder outlet obstruction either anatomical or functional). Courtesy of ICCS (used with permission)

**Fig. 4:**
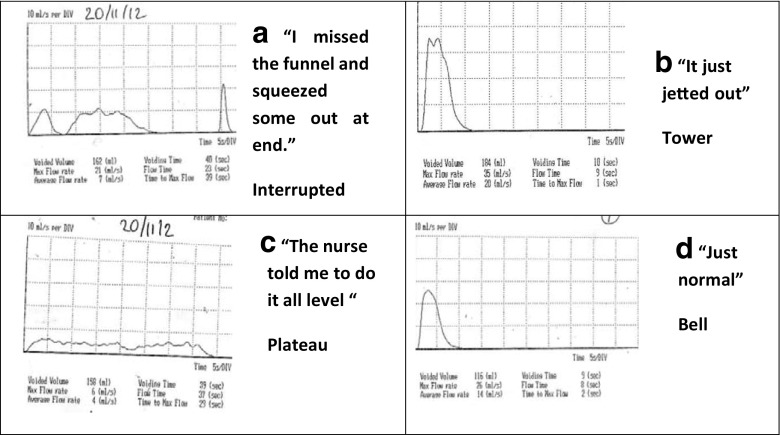
Ten-year-old boy presenting with daytime urinary incontinence with production of four different uroflow curves in single morning demonstrating need for careful interpretation:** a** “I missed the funnel and then squeezed some out at the end”.** b** “It just jetted out”** c** “The nurse told me to do it all level “** d** “Just normal”

Uroflowmetry is a simple, non-invasive and relatively inexpensive tool of investigation that makes it ideal for children. It assesses urinary voiding quantifying volume of urine passed per unit time with maximal or peak flow (q max ml/s) and average flow (q ave. ml/s), as well as voided volume, voiding time, and time to Qmax. The addition of post-void bladder ultrasound scanning using a small hand-held bladder scanner immediately after micturition allows measurement of post-void residual. The equipment is simple to use and most children feel comfortable with it as the uroflowmeter is usually used with a toilet-like commode that can be accommodated in a bathroom or private space allowing representative voiding (see Fig.  7). In order to give accurate results, voided volumes need to be at least 50% of expected bladder capacity (EBC) with a minimum of two adequate volume voids, or preferably three. Overdistention of the bladder should be avoided. Repeated post-void residual volumes of >20mls (4–6 years of age) and >10 ml (7–12 years) are considered abnormal [5]. There are four recognized patterns of uroflowmetry (see Fig. 3, Courtesy of ICCS), although in practice these may not always be easy to interpret or may be mixed. The health professional should always check with the child if the void was representative of what they normally do or if it was different in any way to exclude artefactual information (see Fig. 4). All types of abnormal flow patterns have been reported in healthy children (rates quoted vary from 2.8–37%) but only 3.8% have repetitive nonbell-shaped curves. Whilst there is generally good inter-observer agreement on normal versus abnormal uroflowmetry, the intra- and inter-observer agreement between abnormal uroflow curves is less robust. Nonetheless, uroflowmetry remains a good screening diagnostic tool, particularly in the presence of repeated abnormal flows. There are several nomograms available for peak flow rate (Qmax) with the Tzu Chi nomogram giving a minimally acceptable Qmax of 11.5 ml/s for children <6 years and 15 ml/s for ≥7 years (5–10th centile) [35].

**Fig. 5:**
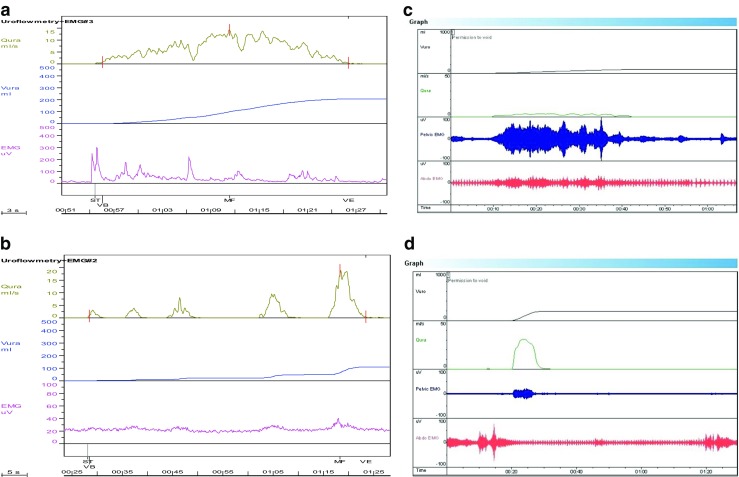
**a** Uroflow (Q ura) showing staccato-shaped curve caused by pelvic floor activity shown in EMG (pelvic): 9-year-old girl presenting with urinary incontinence and UTI.** b** Uroflow (Q ura) showing interrupted-shaped curve with minimal pelvic floor activity on EMG, classic of underactive voiding with abdominal straining: 13-year-old girl presenting with difficulties in voiding.** c** Uroflow (Q ura) showing plateau-shaped curve with significant pelvic floor activity and additional abdominal straining: 10-year-old boy with urinary retention.** d** Uroflow (Q ura) showing bell-shaped curve with accompanying pelvic floor activity and initial abdominal activity that then reduces: 11-year-old girl with difficulties in initiating and maintaining urinary stream

Staccato flow caused by dynamic urethral obstruction during voiding is considered classical of DV. To label flow as staccato the fluctuations should be more than the square root of the maximum flow rate. However, staccato voiding is not exclusive to DV, and one group found that in only one-third of children demonstrating a staccato uroflow was pelvic floor activity the cause as demonstrated by EMG [36]. The same group found that in 121 children shown on EMG to have pelvic floor activity during voiding, uroflows were staccato in 70 (58%), interrupted in 22 (19%), mixed in 12 (10%), and a bell-shaped or depressed curve in 17 (14%) [37]. Interrupted (or fractionated) uroflow is considered classical of underactive detrusor, but alternatively can be caused by complete cessation of flow due to external sphincter closure, and a plateau uroflow usually indicating fixed anatomical outlet obstruction can also be produced by some children with DV. Thus, flow shapes are suggestive only and diagnoses based on uroflow pattern appearance alone can lead to overdiagnoses of dysfunctional voiding and detrusor underactivity. Accompanying simultaneous pelvic floor electromyography is therefore extremely useful for improving diagnostic accuracy. Increased pelvic floor activity can be detected most commonly with surface EMG electrodes rather than needle EMG in pediatrics. This involves the placement of pelvic floor EMG pads usually at position two and ten o’clock around the anus making sure the pads do not connect. This may be embarrassing or uncomfortable for some children and poor pad-skin contact due to wetness or hair can compromise the results. The equipment is often more expensive in addition. It is also true to say that surface EMG electrodes in this position do not accurately reflect external urethral sphincter activity but predominantly levator ani, but at a practical level they represent a reasonable clinical tool. Two channel EMG (pelvic and abdominal) may give extra additional useful information (see Fig. 5).

Where appropriate, further investigation may be required in the form of MRI spinal cord (to exclude neurological causes), cystoscopy (to exclude urethral stricture, posterior urethral valves), videourodynamics (to establish presence of detrusor overactivity, low compliance, bladder neck function, VUR, spinning top urethra), and upper tract assessment.

## Management

**Fig. 6:**
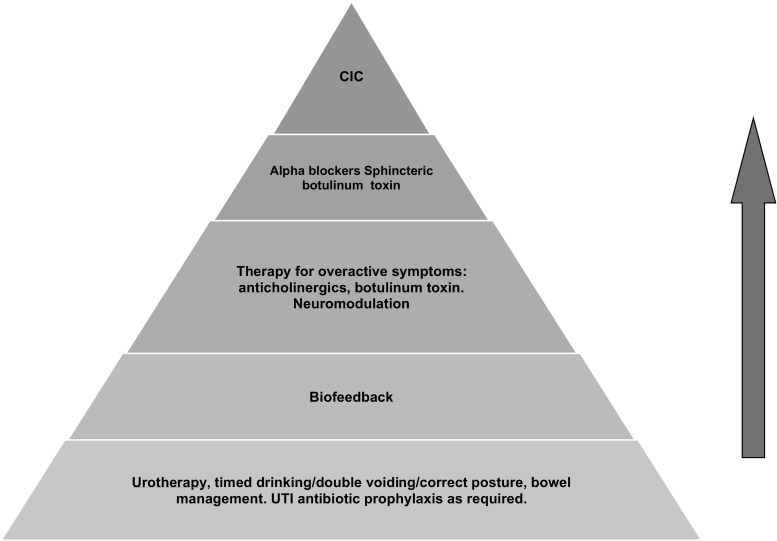
Suggested treatment escalation for dysfunctional voiding in childhood (CIC, clean intermittent catheterization)

Initial steps in management are dependent upon whether there is evidence of damage or significant risk of damage to the kidneys. Those with high risk factors or renal impairment may require additional investigation such as invasive videourodynamics and early institution of clean intermittent catheterization and control of bladder storage pressures. If the risk of damage is low, which is probably in the majority of cases, treatment should have the primary objective to retrain the patient to relax the bladder outlet during voiding with a patient-centric focus. Behavioral therapy is based on the presumption that the disorder is a learned one and hence potentially reversible. It has been shown to be useful in children from the age of 4 years. A proposed management pyramid for children with DV is shown in Fig. 6 and whilst there are no standardized protocols, clinical expert opinion would include these options [10]. We will limit detailed discussion to the use of non-invasive urodynamics used for biofeedback.

The starting point for all childhood LUTD is urotherapy, and there is no exception in DV. The ICCS defines urotherapy as education of the child and parent as to normal bladder and bowel function, a drinking and voiding timetable to include timed voiding/double voiding and adequate fluid intake, correct toileting posture (see below) and aggressive management of constipation [5]. Successful treatment of constipation has been associated with improved voiding parameters [38]. Any behavioral or psychological issues should be identified and addressed. Addition of prophylactic antibiotics to reduce the occurrence of urinary tract infection is often highly successful in helping to break the vicious cycle of dysfunction. Around 20% will have normalization of voiding with this approach [39]. Those not responding should be considered for biofeedback training.

### Non-invasive urodynamics for biofeedback training

**Fig. 7:**
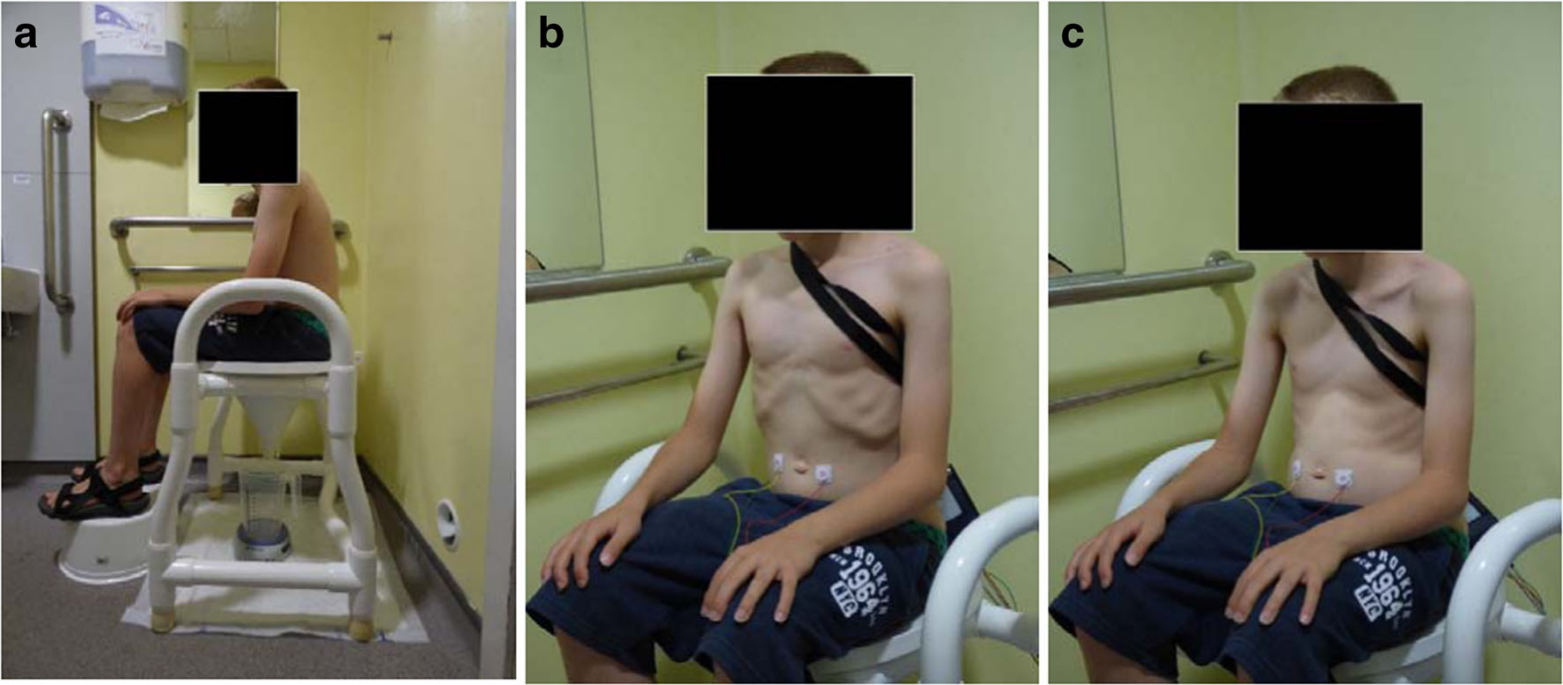
**a** Correct toileting position.** b** Breathing in.** c** Breathing out with abdominal relaxation

**Fig. 8:**
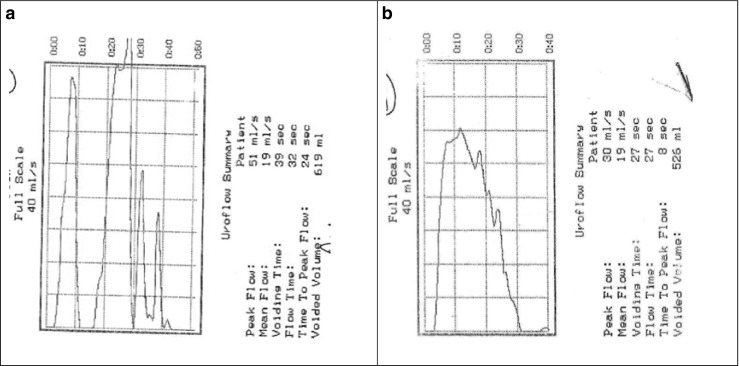
Improved uroflowmetry following simple correct toileting instructions given in outpatient appointment:** a** Fractionated/staccato initial void.** b** Tower/bell-shaped

It is useful as a baseline to get the patient to concentrate on correct toileting posture first and certainly this does not require a specialist therapist and can be taught in a clinic outpatient appointment. This requires the child to be sitting securely on the toilet seat with buttock and foot support with comfortable hip abduction that does not activate abdominal muscle contraction and simultaneous pelvic floor activity. The theory is that the trunk or core muscles are part of an abdominal capsule made up of the pelvic floor, transversus abdominis, the diaphragm and the lumbar spine and multifidus. These muscles act as a continuum and are affected by breathing, lumbar spine positioning, and abdominal activity. In order for the pelvic floor to be maximally relaxed, the trunk must be fully stabilized by proper supported sitting with a fully relaxed abdominal wall, a neutral lumbar spine position (not extended or rounded), and the thighs at the level of the hips, slightly abducted with the feet in a neutral and supported position [40, 41] (see Fig. 7). It is useful to get both the parent and child to participate (using chairs as a pretend toilet in clinic) so that the parent can reinforce the practice at home (see Table 2: Instructions for correct toileting). The effect can be immediate: see Fig. 8.

**Table 2:** Instructions for correct toileting. Encourage parent to participate and reinforce at home

Correct sitting position:
• Sit properly right on top of the toilet, so that the shoulders are over the hips.
• Place both feet flat on the floor or on appropriate step support with knees at the same level as the hips and slightly apart to accommodate at least both fists held together between the knees. The hands can then be placed in a relaxed manner on the thighs.
• Take a deep slow breath in through the nose and slowly out through slightly pursed lips with associated complete relaxation of the abdominal muscles to create a “pot belly” effect. Repeat this and ask the patient to notice what is happening to the pelvic floor when they breathe out and relax their belly (abdomen). Notice that the pelvic floor also bows out and relaxes during exhalation. The visual effect can be reinforced if a mirror is available. Inform the patient that they should only initiate voiding during the relaxed exhalation phase.
During voiding
• If the patient becomes aware of pelvic floor tightening, abdominal straining (or a dip in the uroflow) they should take a short breath in and a long one out again without any abdominal pushing or straining, to re-relax the pelvic floor. Abdominal straining and pushing causes automatic tightening of the pelvic floor.
• Start these steps again if voiding ceases prematurely and attempt to double void.
• At home listen to the sound made by the urinary stream and allow the “gush” of the urinary flow to continue naturally until voiding is complete. Drawing the flow afterwards can reinforce the experience. In Biofeedback training they should watch the uroflow and be invited to describe events afterwards.

Biofeedback is a form of learning or re-education in which the participant is retrained in a normally unconscious physiological process, in this case voiding, via directly tangible signals which may be visual or auditory allowing control and modification of the process and in this case direct relaxation of the pelvic floor. Success of the therapy increases with motivated and adherent patients and enthusiastic, trained therapists and may require multiple sessions. Wennergren and colleagues in 1995 first described physiotherapists working with girls using exercises designed to increase the child’s awareness of their pelvic floor musculature with the focus placed on relaxation as opposed to contraction (which tends to be the focus in adult patients) with improvement described in 12/16 [42]. Since that time, biofeedback has been found to be useful for the immediate and long-term resolution of dysfunctional voiding in numerous studies, with up to 80% sustained effect at 4 years [43]. It has been found to be more effective than urotherapy alone with improvement in voiding pattern, resolution of post void residuals, and reduction in incidence of UTI and VUR. Post-treatment improvement in clinical symptoms correlates with improvement in uroflowmetry parameters and curve patterns. Reduction in symptoms can be achieved regardless of the type of biofeedback used and various methods are available including animated computer programmes and home uroflowmetry [5, 10, 43–45]. Whilst a recent meta-analysis of four studies did not show a significant effect from biofeedback, this was thought by the authors to be due to size and quality limitations [46] and currently it remains expert opinion that it should be a first-line specific treatment after urotherapy, with further good-quality research needed.

Just as uroflowmetry provides the ideal investigation for diagnosis in children due to its simplicity, ease, and non-invasiveness, so it also provides the ideal platform for biofeedback training by having patients view their own voiding curve in real time to identify negative features (staccato dips) and correlate positive features with desired activity. The visual reinforcement can be magnified with auditory reinforcement either with the patient’s own urinary stream or software that links flow to sound. This requires the patient to remain in the hospital for a period of time to produce a number of voids whilst receiving immediate feedback on the uroflow curve.

Some patients also benefit from biofeedback concentrating on pelvic floor muscle isolation and this can be done using perineal EMG surface electrode feedback. There are a number of commercial animated software programmes that are visually engaging for children and with which they generally have positive associations. This can help simplify muscle isolation and encourage patient interest, particularly in those children who are younger or who have attention or motivation issues. The software controller effectively becomes the pelvic floor. The other advantage is that whilst these programmes can be combined with uroflowmetry, they can be carried out independent of voiding uroflowmetry, and this can be time saving. Certainly, EMG has been shown to be as effective as uroflowmetry biofeedback with both animated and non-animated systems. Anecdotally, we find that non-animated systems are easier for older, more cognitively able children, and animated systems are more suited towards younger children or children with developmental difficulties such as autism, learning impairment or, attention deficit hyperactivity disorder. One study has found that animated perineal EMG biofeedback was as efficacious as non-animated, but yielded results more quickly (in 3.6 versus 7.6 sessions) [44].

Real-time ultrasound, either transabdominal at the level of the bladder base or transperineal showing anterior and cranial movement of the bladder neck on contraction of the pelvic floor, with equal attention to relaxation of the pelvic floor, has also been shown to be useful [47].

It is important to measure treatment success and failure using the same tools used to non-invasively evaluate the patient initially including bladder and bowel diaries, uroflow and EMG parameters, post void residual urine measurement, and the recurrence of UTI. Risk factors for failure of biofeedback training include bladder capacity less than 60% of predicted volume and patient noncompliance [45].

Observation during biofeedback often reveals children with pelvic floor dysfunction together with evidence of overactive bladder symptoms who may benefit from anticholinergic medication in conjunction with biofeedback treatment providing they can empty their bladders completely.

## Other treatment considerations

### Alpha blockers

Alpha adrenergic receptors have been demonstrated in the bladder neck and throughout the human urethra and alpha blockers result in decreased bladder outlet resistance with several reports of success in treating children with incomplete bladder emptying [48]. One study compared the results of biofeedback and alpha-blocker medication finding similar reductions in post void residual, but higher parental satisfaction scores with medication. Refractory cases were seen to benefit from a combination of biofeedback and alpha-blocker therapy [49].

### Botulinum toxin-A

Botulinum toxin inhibits acetylcholine release at the presynaptic neuromuscular junction and results in flaccid muscular paralysis and there are several small reports of successful intrasphincteric botulinum toxin-A in the treatment of biofeedback-resistant dysfunctional voiding [50, 51]. Following the injection, it is important to train the child how to void correctly.

### Neuromodulation

Sacral neuromodulation has been used for intractable lower urinary tract dysfunction and in two series in children with DV. The response to neuromodulation (Interstim device) was modest [52] but could be considered in those who have failed other options. One small study used percutaneous tibial nerve stimulation, finding it to be an effective treatment for dysfunctional voiding although chronic stimulation was required to maintain results in one-third of patients [53].

### Surgical

Urethral dilatation has fallen from grace having been found to result in no sustained voiding improvement [15].

### Clean intermittent catheterization (CIC)

CIC allows regular complete emptying of the bladder and is the mainstay of treatment for those high-risk bladders threatening the upper tracts or atonic retentive bladders.

## Summary

Dysfunctional voiding is a voiding disorder associated with failure of the external urethral sphincter to relax during micturition in the absence of neurological or anatomical abnormality, with potential consequences for the entire urinary tract as well as quality of life.

Presentation may be masked by storage symptoms, failure to elicit difficulties with voiding on history, emphasis on urinary tract infections, or vaginal reflux. Diagnosis is made by establishing functional obstructed voiding using non-invasive urodynamics classically (but not exclusively) with a staccato-flow pattern. Urotherapy and non-invasive uroflowmetry, plus or minus surface pelvic floor EMG, provides the mainstay of treatment.

## **Multiple-choice questions** (answers are provided following the reference list)

1. What is the cause of dysfunctional voiding?
  - Neurological impairment
  - Congenital anatomical obstruction of the urethra
  - Failure of pelvic floor and external urethral sphincter relaxation during voiding
  - Small-capacity bladder
2. How might dysfunctional voiding present?
  - Urinary urgency
  - Recurrent urinary tract infections
  - Difficulty initiating urinary stream
  - Weakness in lower limbs
3. How is a suspected case of dysfunctional voiding confirmed?
  - Physical examination
  - Renal tract ultrasound with post void residual measurement.
  - 48-h bladder input-output diary
  - Uroflowmetry +/- EMG
4. Which statement is correct regarding dysfunctional voiding?
  - Commonly leads to kidney failure
  - Can present with secondary urinary incontinence
  - Only occurs in children who have suffered abuse
  - Uncommon condition in childhood
5. Uroflowmetry in dysfunctional voiding:
  - usually occurs with a staccato-shaped flow
  - should only be carried out with two channel EMG
  - may present with any of the known uroflowmetry curves
  - requires the placement of a urethral catheter
6. What is the first-line treatment for dysfunctional voiding?
  - Urotherapy with correct toileting posture
  - Anticholinergic medication
  - Neuromodulation
  - Alpha-blocker medication
7. With regards to correct toileting position
  - Abdominal contraction with inhalation helps relax the pelvic floor
  - Foot support with knees in line with the hips helps relax pelvic girdle muscles
  - A neutral lumbar spine allows relaxation of the pelvic floor
  - Legs should be wide apart with hips tilted forward
